# Diversification and genetic structure of the western-to-eastern progression of European *Phaseolus vulgaris* L. germplasm

**DOI:** 10.1186/s12870-019-2051-0

**Published:** 2019-10-23

**Authors:** Barbara Pipan, Vladimir Meglič

**Affiliations:** Crop Science Department, Agricultural Institute of Slovenia, Hacquetova ulica 17, SI-1000 Ljubljana, Slovenia

**Keywords:** Common bean, SSR markers, Geographic origin, Gene pool, Gene bank, Genetic clusters, Genetic diversity, European accessions

## Abstract

**Background:**

Common bean (*Phaseolus vulgaris* L.) is the most important food legume for direct human consumption around the world, as it represents a valuable source of components with nutritional and health benefits.

**Results:**

We conducted a study to define and explain the genetic relatedness and diversification level of common bean (*Phaseolus vulgaris* L.) germplasm from Portugal to Ukraine, along a western-to-eastern line of southern European countries, including Poland. This was based on the *P. vulgaris* genetic structure, and was designed to better describe its distribution and domestication pathways in Europe. Using the multi-crop passport descriptors that include geographic origin and different phaseolin types (corresponding to the Mesoamerican and Andean gene pools), 782 accessions were obtained from nine gene banks and 12 geographic origins. We selected 33 genome/ gene-related/ gene-pool-related nuclear simple sequence repeat markers that covered the genetic diversity across the *P. vulgaris* genome. The overall polymorphic information content was 0.800. Without specifying geographic origin, global structure cluster analysis generated 10 genetic clusters. Among the *Pv*SHP1 markers, the most informative for gene pool assignment of the European *P. vulgaris* germplasm was *Pv*SHP1-B. Results of AMOVA show that 89% of the molecular variability is shared within the 782 accessions, with 4% molecular variability among the different geographic origins along this western-to-eastern line of southern Europe (including Poland).

**Conclusions:**

This study shows that the diversification line of the European *P. vulgaris* germplasm followed from the western areas of southern Europe (Portugal, Spain, Italy, Slovenia) to the more eastern areas of southern Europe. This progression defines three geographically separated subgroups, as the northern (Poland, Ukraine, Romania), southern (Albania, Bulgaria), and central (Bosnia and Herzegovina, Serbia, Hungary) areas of eastern Europe.

## Background

The common bean (*Phaseolus vulgaris* L.) is the most important food legume throughout the world for direct use (for review, see [1]). *P. vulgaris* is also a rich source of polyphenolic compounds that have numerous health-promoting properties [2].

In the last decade, several thousand accessions of *Phaseolus* have been collected ever more intensively in different parts of Europe, and these are stored in national gene banks. At present, the *Phaseolus* database in the web-based European Search Catalogue for Plant Genetic Resources (EURISCO) contains over 50,000 records, which includes more than 40,000 characterisation and evaluation records, and 585 photographs [3]. Due to the distributions of the wild forms, a number of studies have suggested that *P. vulgaris* has gone through at least two isolated and independent domestication processes. Consequently, two independent events in the Americas have been documented (i.e., in Mesoamerica and the Andes) [1, 4, 5], from where the two major domesticated gene pools originated (for review, see [6]).

Phaseolin is the major seed storage protein of common bean, and it is an important molecular marker [7]. For Mesoamerican origin, the specific types of phaseolin are Sanilac, as S type, Middle America, as M type, and Boyacá, as B type [7, 8]. The predominant phaseolin type for the Andean gene pool is Tendergreen, as T type, followed by Contender, as C type, which can also indicate a mixed origin with the Mesoamerican form. Huevo de Huanchaco, or H type, phaseolin also belongs to the Andean group. The three indel spanning markers SHP1-A, SHP1-B and SHP1-C were used for identification of the gene pool of origin, as Nanni et al. [9] reported that for 91 *P. vulgaris* accessions, no Ib haplotypes were shared between the Mesoamerican and Andean gene pools.

Based on evaluations of morphological variation and microsatellite diversity, Rendón-Anaya et al. [10] and others [11–14] reported that the accompanying domestication processes, such as increased genetic diversity of the domesticated varieties, arose from domestication bottlenecks and hybridisation events between the wild and domesticated populations. The wild progenitor of common bean has an exceptionally large distribution, from northern Mexico to north-western Argentina, which is unusual among wild progenitors of today’s crops [15]. These same recent studies also report on the molecular ecology, selection and adaptation in the drought-related genes/ polymorphisms in wild and cultivated common bean [16–18]. Moreover, two recent studies have reported that the genome-wide association in wild common bean predicts widespread divergent adaptation to drought [19, 20], and that species divergence in *Phaseolus* beans has led to parallel signatures of adaptation and domestication [21]. Genetic diversity assessments and the structure of *Phaseolus coccineus* L. clearly differentiate between the European and Mesoamerican gene pools, which infers a moderate to strong cytoplasmic bottleneck that followed the expansion of *P. coccineus* into Europe through multiple domestication events [22].

For European *P. vulgaris* germplasm, Carović-Stanko et al. [8] reported that the main group of European accessions were of Andean origin (~ 68%), with fewer of Mesoamerican origin (~ 27%). The rest of the European accessions represented putative hybrids between these two gene pools. Additionally, Maras et al. [23] indicated that Andean genotypes were more prevalent than Mesoamerican in the countries from the five former Yugoslav republics (except for Macedonia) that constituted the western Balkans. This kind of trend might be the consequence of the political regulation within these countries in the past. Both of these studies used combinations of molecular and seed-protein markers. However, simple sequence repeat (SSR) markers have also been shown to be one of the most informative, efficient and cost-effective tools for population genetics studies of different agronomically important plant species [23–32]. Recently, Campa et al. [33] published a study on Spanish *P. vulgaris* diversity (308 local lines, mainly used for snap consumption), where they indicated that 70% of the lines were associated with the Andean gene pool. The panel was characterised by 3099 single nucleotide polymorphism (SNP) markers that were obtained through genotyping-by-sequencing, which revealed wide genetic diversity and low levels of redundant material within the panel [33]. Moreover, the genetic diversity and population structure in the common bean in Turkey was examined using diversity arrays technology (DArT) markers, which revealed two main populations, as A (predominant) and B, and five unclassified genotypes. These represented three meaningful heterotic groups for breeding purposes [34]. Thus, current advances in molecular technologies can provide evidence of the human selection that has acted on numerous loci during and after crop domestication [35]. Moreover, with its different adaptation abilities, common bean might be suitable for organic farming systems [36].

In the present study, we genotyped 782 accessions from 12 (country-based) areas of geographic origin from Portugal to Ukraine, along a western-to-eastern line of southern European countries (including Poland) using 33 loci: 24 markers for diversity levels; three markers related to gene pool assignment; and six gene-related markers. The main objectives of this study were: (i) to identify and collect the most geographically different *P. vulgaris* accessions from these European areas; (ii) to obtain global diversification levels and the genetic structure of *P. vulgaris* from these European areas without specifying geographic origin; (iii) to define the genetic relatedness of *P. vulgaris* accessions from these European areas in terms of their geographic origins; (iv) to detect allelic diversity of *Pv*SHP1 markers related to gene-pool assignment of the germplasm from these European areas; and (v) to identify the genetic potential of the germplasm from these European areas for association mapping studies, to help with the identification of new sources of genetic diversity. The data from the present study can be used mainly to investigate the levels of genetic diversity and the genetic structure of common bean from these 12 European areas, and to acquire new knowledge about its expansion processes and related diversification in Europe. This knowledge can now be used for common bean breeding, especially in terms of its adaptation to different environments.

## Results

### Geographic origins of the genotyped accessions

On a basis of the initial screening using multi-crop passport descriptors, the full set of samples comprised 782 accessions from 12 geographic origins: Albania (23), Bosnia and Herzegovina (57), Bulgaria (19), Hungary (277), Italy (20), Poland (18), Portugal (8), Romania (14), Serbia (218), Slovenia (97), Spain (11) and Ukraine (20) (Additional file 4: Table S1; numbers of assessed accessions within each country are included in Fig. 4b). For 63 of the accessions from Slovenia, Italy, Portugal and Spain, the information on phaseolin type included C type (23), B type (1), H type (1), S type (18) and T type (20), which served as an orientation for the gene-pool assignment (Additional file 4: Table S1).

### Diversification level and genetic structure for the western-to-eastern line of southern European *P. vulgaris* germplasm

Within the whole set of 782 accessions, the overall number of alleles with frequency ≥ 5% was 5.39, and the number of private alleles was 27.52. All of the SSR markers were polymorphic, and the whole set of 782 accessions showed statistically significant *HWE* deviation for all loci (*p* < 0.001). Moreover, combining genetic variability parameters among loci and other measures of population genetics did not indicate any deviations that might be due to a significant frequency of null alleles. The genetic variability defined by the expected heterozygosity (*H*_*e*_) for the whole collection was 0.822. Overall the *PIC* reached 0.800, with a mean of 6.842 effective alleles per locus, and a mean of 22.767 for *Ar* (Table 1). The most informative SSR markers (i.e., with *PIC* > 0.9) were GATS91 (*PIC* = 0.923; *H*_*e*_ = 0.928; *Ne* = 13.749), BMd001 (*PIC* = 0.917; *H*_*e*_ = 0.923; *Ne* = 12.801), ATA006 (*PIC* = 0.905; *H*_*e*_ = 0.912; *Ne* = 11.346) and SSR-IAC62 (*PIC* = 0.925; *H*_*e*_ = 0.930; *Ne* = 14.137), which defined the highest *H*_*e*_ (> 0.9) and the highest *Ne* (> 11). For these loci, *Ar* was high (> 23). Locus BMd001 had the overall highest *Ar* (43.451) and *I* (2.944). The highest *F* was obtained for ATA007 (0.663), and the lowest *F* for locus BM210 (− 0.794). On the other hand, the least polymorphic loci were SSR-IAC167 and BM210, which had the lowest *H*_*e*_ (0.585; 0.540; respectively), *PIC* (0.542; 0.434), *Ne* (2.405; 2.172) and *I* (1.215; 0.867) (Table 1).

**Table 1:** Parameters of the genetic variability of the 782 accessions from the western-to-eastern areas of southern Europe, across the 33 loci

Locus	Expected heterozygosity (*H*_*e*_)	Polymorphic information content	Allelic richness (*Ar*)	Number of effective alleles (*Ne*)	Shannon’s information index (*I*)	Fixation index (*F*)
ATA003^*a^	0.879	0.867	27.709	8.212	2.446	0.198
ATA004^*a^	0.849	0.834	24.000	6.551	2.326	0.362
ATA005^*a^	0.843	0.828	30.132	6.362	2.392	−0.040
ATA007^*a^	0.804	0.779	24.845	5.080	2.040	0.663
ATA016^*a^	0.790	0.762	17.147	4.752	1.873	−0.066
GATS91^*c^	0.928	0.923	32.292	13.749	2.890	−0.060
ATA002^*a^	0.858	0.845	30.798	6.982	2.452	0.044
BM172^*c^	0.894	0.885	35.118	9.411	2.686	0.097
BMd001^*b^	0.923	0.917	43.451	12.801	2.944	0.019
ATA020^*a^	0.882	0.872	34.052	8.422	2.596	0.288
Pv-ag004^*i^	0.845	0.828	26.295	6.432	2.287	−0.100
SSR-IAC167^**h^	0.585	0.542	12.735	2.405	1.215	0.116
ATA010^*a^	0.825	0.802	14.617	5.690	1.986	0.435
BM155^*c^	0.741	0.694	9.139	3.847	1.447	−0.168
BM170^*c^	0.889	0.879	27.770	8.960	2.537	−0.060
BM183^*c^	0.724	0.682	14.747	3.610	1.601	−0.285
BM210^*c^	0.540	0.434	5.402	2.172	0.867	−0.794
BMd044^*b^	0.762	0.726	13.088	4.187	1.667	0.236
ATA009^*a^	0.901	0.893	23.516	10.083	2.593	0.179
ATA145^*a^	0.796	0.781	22.613	4.888	2.162	0.431
GA16^*a^	0.855	0.837	15.225	6.858	2.110	0.090
ATA006^*a^	0.912	0.905	23.074	11.346	2.642	0.085
BM157^*c^	0.813	0.788	14.464	5.345	1.938	0.461
BMd042^*b^	0.857	0.842	24.054	6.977	2.312	−0.104
ATA289^*a^	0.773	0.743	20.694	4.391	1.861	−0.239
PvSHP1-A^***d^	0.855	0.839	17.111	6.868	2.195	−0.138
PvSHP1-B^***d^	0.856	0.838	16.937	6.911	2.093	−0.132
PvSHP1C^***d^	0.849	0.833	16.159	6.615	2.173	−0.004
PvM04^**f^	0.734	0.705	14.745	3.752	1.727	0.359
PvM21^**f^	0.753	0.724	17.757	4.047	1.752	−0.279
PvM95^**g^	0.779	0.752	28.173	4.511	1.980	−0.208
SSR-IAC62^**e^	0.930	0.925	32.777	14.137	2.892	0.073
SSR-IAC66^**e^	0.895	0.885	40.677	9.449	2.671	−0.061
Mean values	0.822	0.800	22.767	6.842	2.162	0.042

^*^marker to assess diversity levels; ^**^trait related marker; ^***^ marker related to gene pool assignment; ^a^Blair et al., [37]; ^b^Blair et al. [38]; ^c^Gaitan-Solis et al. [39]; ^d^Nanni et al. [9]; ^e^Benchimol et al. [40]; ^f^Hanai et al. [41]; ^g^Hanai et al. [42]; ^h^Campos et al. [43]; ^i^Yu et al. [44]

**Fig. 1:**
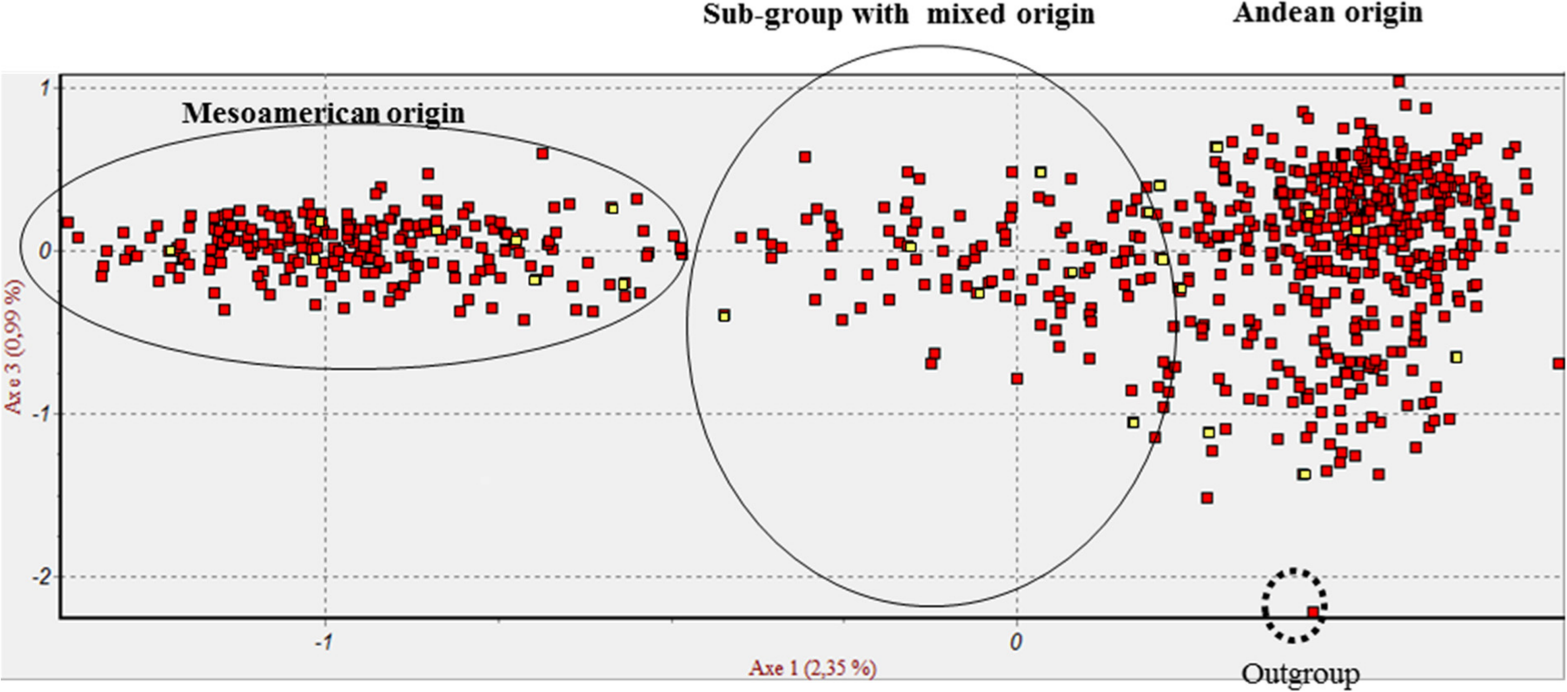
Global factorial correspondence analysis distribution of the accessions from the western-to-eastern areas of southern Europe. Yellow symbols, accessions with known phaseolin type within each group

The global distribution in the FCA for these accessions from western-to-eastern areas of southern Europe defined four factors with a 5.35% level of integrity. In general, three main groups of accessions were defined, which corresponded to the Mesoamerican and Andean subgroups, plus a subgroup with mixed origins, and an out-group that only included the *P. coccineus* accession (Fig. 1). To link the membership of each group, the standard accessions with a known type of phaseolin were each used for orientation. Therefore, the accessions with phaseolin T type were assigned to the typical Andean gene pool, the accessions with phaseolin S type to the typical Mesoamerican gene pool, and the accessions with phaseolin C type were the members of the subgroup with mixed origins extracted from the Andean subgroup and leading to the Mesoamerican subgroup (Fig. 1, yellow symbols within each cluster). Based on the phylogenetic relationships between the whole set of accessions (Additional file 2: Figure S2), analysis of the three main groups was carried out according to Nei’s standard genetic distance [45] and the UPGMA clustering method.

When the Bayesian/ Evanno approach was applied without specifying the population of origin, 10 genetic clusters were generated from the admixture model for the 782 accessions (Additional file 1: Figure S1). The means of the calculated genetic distances among these 10 clusters were from 0.681 to 0.812, which corresponded to F statistic (*Fst*) from 0.013 to 0.150.

### Genetic relatedness among the different geographic origins

Determination of the genetic relatedness among the 12 geographic origins was performed on the basis of the data obtained for all 33 of the loci. The results of the cluster analysis using the information on the gene-bank origins of the accessions showed that there were only three genetic clusters, which showed genetic diversities (across the genome diversity) of 0.749, 0.738 and 0.772. Moreover, the molecular variability of the *P. vulgaris* accessions among nine gene banks was 3%, while the other 97% of the *P. vulgaris* molecular variability of the gene pools did not account for significant structure (AMOVA, *p* > 0.010) (Additional file 6: Table S3).

Considering the information about the geographic origins (i.e., the 12 countries) instead of the gene-bank origins, seven genetic clusters were generated using the Bayesian/ Evanno algorithm (Additional file 3: Figure S3). To clarify these analyses, the same algorithm and the same criteria for choosing the optimum number of clusters was used in each, but with different original information given in the input matrix (i.e., without specifying the population of geographic origin [only one/ European origin]; using the information of the gene-bank origins [eight gene-bank origins]; and specifying the geographic origin [12 geographic origins]); these thus provided different results (i.e., different numbers of genetic clusters). The numbers of genetic clusters that were generated on the basis of the genetic structure with the specification of the population of origin of the accessions varied from three (Hungary) to 11 (Italy, Poland) (Table 3). The mean distances between the accessions in the same clusters varied from 0.669 to 0.808. The results of the AMOVA showed that 89% of the molecular variability was shared within the 782 accessions, and that there was only 4% molecular variability among the different geographic origins, which did not define significant structure from these western-to-eastern areas of southern Europe (Table 2).

**Table 2:** Analysis of molecular variance considering the geographic origins of the accessions

Source	Degrees of freedom (df)	Sum of the squared differences	Mean square	Estimated variance	Percentage of molecular variance
Among geographic origins	11	749.415	68.129	0.490	4^*^
Among accessions	770	10,987.300	14.269	1.081	8
Within accessions	782	9467.500	12.107	12.107	89
Total	1563	21,204.215		13.678	100

^*^p < 0.01 (F-statistics)

**Fig. 2:**
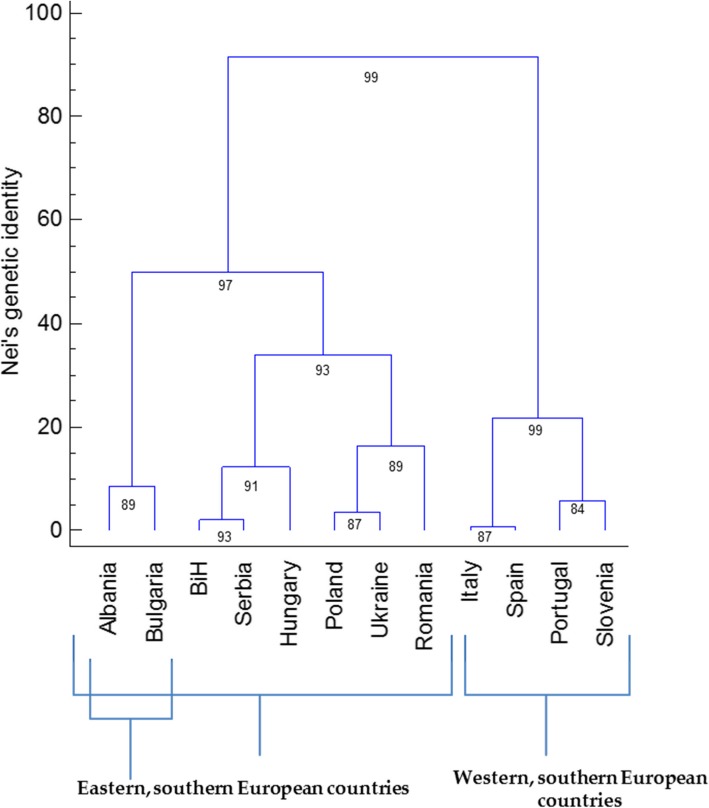
Squared Euclidian dendrogram of the geographic relatedness according to the pairwise matrix of Nei’s genetic identity and Ward’s method with bootstrap support. BIH, Bosnia and Herzegovina

Regarding the pairwise comparisons of accessions within each geographic origin, the highest Nei’s genetic identities were seen both between neighbouring countries, such as Poland and Ukraine (0.882) and Bosnia and Herzegovina and Serbia (0.892), and between non-neighbouring countries, such as Italy and Spain (0.810) (Fig. 2). The estimation of the gene flow among the geographic origins through the private allele method of Slatkin [46] was 0.541, and the corrected estimated value of Barton and Slatkin [47] was 0.401.

The accessions that originated from Hungary, Serbia and Slovenia did not show any deviations from *HWE* for any of the loci. The highest levels of genetic variability were seen for the Hungary, Italy, Poland and Slovenia accessions (*uH*_*e*_ > 0.8). Considering *Ar*, the accessions from Serbia reached the higher level of fundamental genetic variation that is suitable for conservation (*Ar* = 1.678). Correspondingly, the highest proportion of molecular variability explained for PCoA (with the first three axes) was for the Serbia accessions (73.5%), followed by the accessions from Bosnia and Herzegovina (71.0%). Moreover, PCoA was performed within each country, and the proportions of explained genetic variability with the first three axes are given in Table 3. Regardless of the number of genetic clusters defined for each geographic origin, the mean *H*_*e*_ across the clusters for each origin varied from 0.676 for the Albania accessions, to 0.809 for the Italy and Serbia accessions (Table 3). The loci that were without statistically significant deviations from HWE (*p* > 0.05) are given in Additional file 7: Table S4.

**Table 3:** Geographic details and genotypic summary statistics of the accessions for the 33 loci

Geographic origin	Number of accessions	Allelic richness (*Ar*)	Number of genetic clusters (real *K*)	Expected heterozygosity (*H*_*e*_) across clusters	Allele frequency ≥ 5%	Global unbiased expected heterozygosity of accessions	Principle coordinate analysis (first three axes) (%)	Mean polymorphic information content among all loci
Albania	24	6.369	5	0.676–0.681	3.515	0.626	67.7	0.569
Bosnia and Herzegovina	56	7.690	6	0.739–0.805	4.939	0.783	71.0	0.746
Bulgaria	19	1.794	7	0.778–0.779	5.788	0.794	69.3	0.724
Hungary	277	16.653	3	0.747–0.764	5.242	0.815	68.5	0.815
Italy	20	4.699	11	0.808–0.809	6.061	0.815	65.6	0.761
Poland	18	4.066	11	0.805–0.807	6.273	0.816	63.6	0.761
Portugal	8	1.791	9	0.745–0.747	5.909	0.791	68.2	0.683
Romania	14	6.330	5	0.754–0.755	5.091	0.746	62.8	0.725
Serbia	218	16.678	9	0.804–0.809	5.121	0.797	73.5	0.769
Slovenia	97	10.405	5	0.788–0.774	5.152	0.816	69.0	0.788
Spain	11	1.786	8	0.787–0.789	5.030	0.786	66.0	0.715
Ukraine	20	1.793	10	0.772–0.773	5.939	0.793	63.7	0.723

Considering the mean *PIC*, the applied set of 33 SSR markers was the most informative for the Hungary accessions (0.815), and the least informative for the Albania accessions (0.569) (Table 3).

### Allelic diversity of PvSPHP1 markers related to the gene-pool assignments

**Fig. 3:**
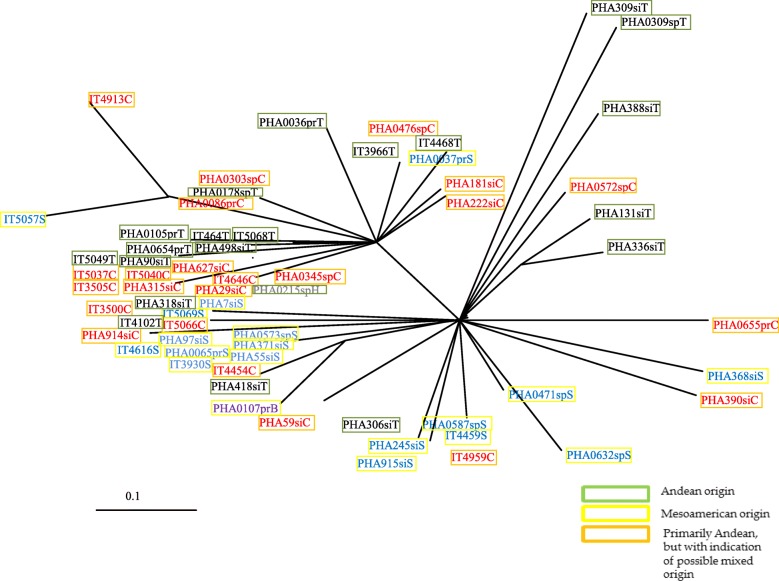
Genetic relatedness (as *Ds*; unweighted pair group method with arithmetic mean) of the accessions with known phaseolin types for the *Pv*SHP1 loci (as indicated by last letter in different colours). Geographic origin: si, Slovenia; sp., Spain; pr, Portugal; it, Italian geographic origin. *Ds*, Nei’s standard genetic distance

Based on the known information about phaseolin types, 63 accessions were screened in detail at the *Pv*SHP1-A, *Pv*SHP1-B and *Pv*SHP1-C loci, to determine whether there were any significant patterns related to the phaseolin types or the gene-pool assignments. In general, these three loci deviated from *HWE* for the groups of all three of the main phaseolin types (i.e., C, S, T), with statistical significance seen (*p < 0.01*), except for PvSH1-C within the phaseolin T and S groups*.* Here, these markers can be used to distinguish between the phaseolin T and S types. In general, the accessions with phaseolin C or S types and phaseolin C or T types were clustered together, except for accessions IT4102T, PHA0107prB, PHA418siT and PHA318siT (Fig. 3).

**Fig. 4:**
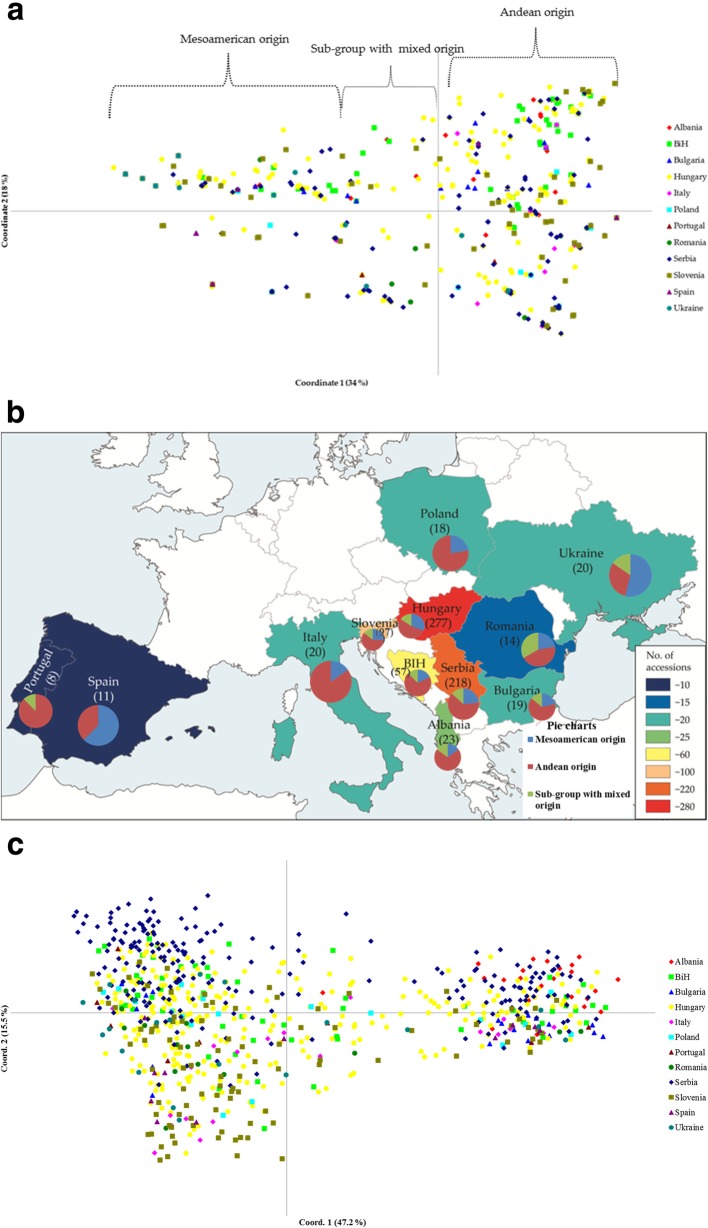
**a** Principal coordinate analysis distribution for the *Pv*SHP1 markers for the accessions from the 12 geographic origins. **b** Map with pie charts showing breakdown of the three sub-groups for each country on the basis of *Pv*SHP1 markers including number of assessed accessions within each geographic origin (in brackets). Source of map: https://www.morevectors.com/administrative-europe-map-vector/. **c** Global principal coordinate analysis distribution for all 33 loci for the accessions from the 12 geographic origins

For the geographic origins, no significant deviations from *HWE* were seen for the marker *Pv*SHP1-C for the Bulgaria, Albania and Portugal accessions, and for the marker *Pv*Sh1-A for the Spain accessions. The distribution pattern along the *Pv*SHP1 markers generated two groups; from the main group of Andean origin (Fig. 4a, right) through the subgroup with mixed origin, to the main group of Mesoamerican origin (Fig. 4a, left), thus combining the accessions from all of the screened geographic origins (Fig. 4c).

As seen from Fig. 4a, the accessions from all of the geographic origins were distributed along the first two principal coordinates, with sharing of the same *P. vulgaris* germplasm from Andean to Mesoamerican, and to the subgroup with mixed gene pools. Detailed information on the basis of the *Pv*SHP1 markers and PCoA distribution are presented in Fig. 4b, where the 12 pie charts superimposed on the geographic map show the breakdown of the three subgroups for each country. The background of Fig. 4b is keeping the information about the number of accessions (in brackets), to better present the analysed accessions from each geographic origin. As seen from Fig. 4b, the Andean origin is predominant in 10 European countries. The lowest level of Andean genotypes was for Spain (37.5%) and Ukraine (30.8%), with the highest for Italy (84.6%) and Portugal (87.5%). On average, the European *P. vulgaris* germplasm from the 12 geographic origins was 62% of Andean origin, 25.8% of Mesoamerican origin and 12.2% of mixed origin (summarised from Fig. 4b).

For comparative purposes, the global PCoA distribution for all 33 loci for the accessions from the 12 geographic origins are presented in Fig. 4c. The first three axes for the PCoA cumulatively explain 73.5% of the genetic variability (Fig. 4c; data shown for first two axes only).

**Fig. 5:**
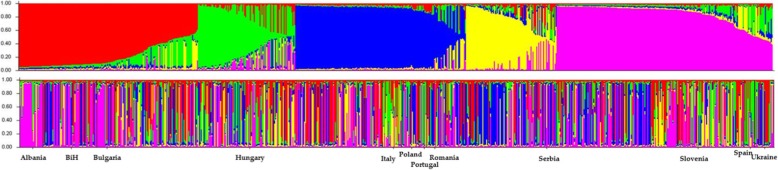
Global structure plot of the accessions sorted by Q value (top), and for their genetic structure sorted by geographic origin (bottom) on the *PvSh1* loci. BIH, Bosnia and Herzegovina. Each vertical bar represents the individual genotype; the colour within each bar represents the colour of different genetic cluster

The global structure analysis for the geographic origins on the *Pv*Sh1 loci formed five genetic clusters (0.623 ≤ *H*_*e*_ ≥ 0.8028) that joined the accessions from the mixed origins (attributed to the most likely genetic group of origin according to the highest Q value in a structure plot) (Fig. 5).

### Genetic potential for association mapping studies

Four loci suitable for association mapping (*Pv*M04, *Pv*M21, SSR-IAC62, SSR-IAC66) and two loci related to angular leaf spot/ anthracnose resistance (*Pv*M95, SSR-IAC167) were included in the present study. Ten genetic clusters were formed here, with the accessions either subdivided into the geographic groups of the 782 accessions (0.7808 ≤ *H*_*e*_ ≥ 0.7951) or not (0.8308 ≤ *H*_*e*_ ≥ 0.8437). No statistically significant deviations from *HWE* (*p > 0.05*) were seen for loci *Pv*M04, *Pv*M21 and SSR-IAC167 for the Italy accessions, *Pv*M21, SSR-IAC62 and *Pv*M95 for the Poland accessions, *Pv*M04, *Pv*M21, *Pv*M95 and SSR-IAC66 for the Portugal accessions, and *Pv*M21 and *Pv*M95 for the Spain accessions.

The highest numbers of alleles with a frequency ≥ 5% were detected within the Portugal (6.500), Poland (6.500), Italy (6.333) and Bulgaria (6.167) accessions. The highest numbers of effective alleles were obtained for the Poland (*Ne* = 6.227) and Hungary (*Ne* = 6.155) accessions. The lowest numbers of both allele frequency ≥ 5% (4.000) and *Ne* (3.382) were seen for the Albania accessions. No private alleles were detected within the Ukraine, Romania and Portugal accessions. In contrast, the highest numbers of private alleles (*Np*) were seen for the Hungary (3.667), Serbia (2.500) and Slovenia (1.667) accessions, which corresponded to the highest numbers of accessions included. The highest numbers of locally common alleles were seen for the Slovenia (4.167), Hungary (4.000), Serbia (3.500), Poland (3.167) and Bosnia and Herzegovina (3.167) accessions. According to the trait-associated markers, the least genetically diverse were the accessions from Albania (*H*_*e*_ = 0.648; *uH*_*e*_ = 0.663), Romania (*H*_*e*_ = 0.709; *uH*_*e*_ = 0.736) and Bosnia and Herzegovina (*H*_*e*_ = 0.712; *uH*_*e*_ = 0.719). The accessions with the highest diversity potential for association mapping studies were from Italy (*H*_*e*_ = 0.812; *uH*_*e*_ = 0.834), Spain (*H*_*e*_ = 0.771; *uH*_*e*_ = 0.809) and Slovenia (*H*_*e*_ = 0.793; *uH*_*e*_ = 0.809) (Table 4). These statistics and the data in Table 4 were calculated for the accessions that originated from each country/ geographic origin and are not compatible with the genetic clusters calculated on the basis of the genetic structure of all of the origins together. Here, we calculated the allelic pattern(s) within each country to compare the population statistics parameters among these European countries, not to compare the genetic clusters conducted from these European countries. So, these analyses follow different points of view in terms of the data presented: on a global scale with no prior information (Fig. 1) or with prior information on the geographic origins (Fig. 2), or analysis of the allelic patterns on the segmented/ national levels separately for each country (Table 4). Moreover, Table 3 summarises the data for the trait-related loci only, to show the complete data and geographic details for all 33 loci, as calculated for each country separately.

**Table 4:** Allelic patterns (main values) across the accessions from the different geographic origins for the trait-related loci

Geographic origin	Allele frequency ≥ 5%	Number of effective alleles (*Ne*)	Shannon’s information index (*I*)	Number of private alleles (*Np*)	Number of locally common alleles		Expected heterozygosity (*H*_*e*_) across clusters	Expected unbiased heterozygosity (*uH*_*e*_) across clusters
					≤25%	≤50%		
Albania	4.000	3.382	1.424	0.833	0.667	2.000	0.648	0.663
Bosnia and Herzegovina	4.000	4.964	1.728	1.500	1.167	3.167	0.712	0.719
Bulgaria	6.167	5.855	1.722	0.167	0.667	2.000	0.767	0.787
Hungary	4.667	6.155	1.941	3.667	1.667	4.000	0.757	0.758
Italy	6.333	5.967	1.920	0.333	1.333	3.000	0.812	0.834
Poland	6.500	6.227	1.819	0.333	0.833	3.167	0.762	0.784
Portugal	6.500	4.697	1.560	0.000	0.833	2.167	0.723	0.771
Romania	5.167	4.543	1.560	0.000	0.833	1.833	0.709	0.736
Serbia	4.833	5.440	1.869	2.500	1.333	3.500	0.762	0.764
Slovenia	4.833	5.802	2.000	1.667	1.833	4.167	0.793	0.797
Spain	4.500	4.900	1.754	0.833	1.167	2.333	0.771	0.809
Ukraine	5.667	4.951	1.667	0.000	0.833	2.500	0.724	0.743

## Discussion

The first major achievement in this study was to obtain a high number of highly diverse accessions that represented the core *P. vulgaris* germplasm from western-to-eastern areas of southern Europe. Considering the criteria used for the selection (i.e., geographic origin, biological status, ancestral data, phenotypic seed characteristics, phaseolin type), a collection of 782 accessions from 12 geographic origins was formed. This represents a rich source of agronomically important traits that have adapted to European growth conditions.

Our data show that high levels of *P. vulgaris* genetic diversity are maintained within the European gene banks. Three genetic clusters were identified within the *P. vulgaris* accessions kept in nine gene banks, which corresponded to three subgroups considering the main *P. vulgaris* origins: Mesoamerican, Andean and a subcluster with mixed origins. To confirm this, the mean diversity levels between the accessions within each subcluster only varied from 0.738 to 0.772. The nine European gene banks included in the study are actually preserving the *P. vulgaris* germplasm according to the relative equality of the Mesoamerican and Andean gene pools. In contrast, 10 genetic clusters were generated when none of the information on the gene banks or the geographic origins was used to subdivide the accessions. The key point for interpretation of the results on the numbers of clusters from different observations is the different prior input information and data processing for the genetic structure analyses. The data for both of these cluster analyses revealed high levels of genetic diversity throughout the whole set of these 782 European accessions. *H*_*e*_ among the accessions within each gene cluster varied from 0.6814 to 0.8116, which comprised the Mesoamerican and Andean subclusters, plus the subcluster with putative hybrids from both of these gene pools. This distribution was confirmed by the FCA. The key point of the FCA was to define the overall/ general genetic diversity among the individuals summarised using the multivariate method through presentation of the distribution pattern of the 782 accessions without any prior information (such as phaseolin type, phylogenetic relations). This FCA generated three main groups that were assigned as Mesoamerican, Andean and mixed on the basis of the standard accessions with known types of phaseolin (63 accessions), as an orientation point to classify which group is which. This FCA has no direct connection with the phylogram in Additional file 2: Figure S2, which was performed on the basis of the genetic relationships (i.e., Nei’s standard genetic distances) combined with the UPGMA clustering method, thus with a different algorithm used compared with the FCA. Here we see in particular that these two different algorithms generated three main groups that can be defined as Mesoamerican, Andean and mixed (not assigned in Additional file 2: Figure S2, with the aim being to illustrate the phylogenetics of the whole 782 accessions). To compare these data to previous studies, Maras et al. [23] described two large clusters that corresponded to two gene pools of origin for accessions from the western Balkans. The comparison in the present study indicates that the accessions from both the western and eastern parts of Europe introduced different alleles, which thus increased the *P. vulgaris* diversity. This is also reflected by the third subgroup for the molecular data for the present study, which showed mixed Mesoamerican and Andean origins.

### Marker diversity and their applicability within this western-to-eastern European *P. vulgaris* collection

The set of 33 SSR markers used in the present study have been shown to be polymorphic, species-specific and informative for distinguishing polymorphisms through the Mesoamerican and Andean germplasm [9, 37–44]. These markers have different repeat motifs and cover all of the linkage groups of the *P. vulgaris* genome. In general, the selected set of SSR markers was highly applicable and proved useful to detect the high diversification levels of these European accessions (*PIC* = 0.800; *H*_*e*_ = 0.822) and to define the gene-pool structure. Indeed, when looking at data from a previous study, the overall *PIC* (as a general measure of marker diversity) of 104 wild *P. vulgaris* accessions and 606 cultivated genotypes using 36 loci (as a combination of both genomic and gene-based markers, as in the present study) was 0.64 (as calculated for wild and cultivated accessions) [48]. In comparison, when 13 loci were used, the germplasm from the western Balkans related to a western-to-eastern European line was more genetically uniform, as seen by the lower values of the diversity parameters among the loci (*PIC* = 0.72; *H*_*e*_ = 0.76) [16]. Moreover, based on 26 SSR loci, *H*_*e*_ for Croatia landraces was reported as 0.572 [7]. Then for a set of 123 SSR markers (SSR-IAC series), the *PIC* varied from 0.05 to 0.83 when screened for 20 *P. vulgaris* cultivated genotypes [40]. The high *Ar* in the present study (mean, 22.767) as a fundamental measure of the genetic variation reflects the heterogeneity of the European common bean germplasm. Consequently, the *F* might indicate the undetected null alleles (e.g., at loci ATA016, BM157) resulted in excess heterozygosity (e.g., at locus BM210). The set of SSR markers applied here was most informative for the Hungary and Slovenia accessions. Similarly, for the Hungary, Portugal, Serbia and Slovenia accessions only, all of the loci showed significant deviation from *HWE* (*p < 0.05*). This situation indicates that a set of these 33 loci would be applicable to effectively investigate the *P. vulgaris* germplasm.

### Genetic structure and level of diversification among this western-to-eastern European *P. vulgaris* germplasm

Despite the high levels of genetic variability of the *P. vulgaris* germplasm in the present study (i.e., 10 genetic clusters), only 4% of the molecular variability was found among the 12 geographic origins from these western-to-eastern European areas, with 8% among the accessions and 89% within the accessions, and with 97% of the molecular variability maintained within the European gene banks.

The present study shows that on the basis of Nei’s genetic identity matrix (for pairwise comparisons) [454], the diversification line of the European *P. vulgaris* germplasm follows from the western parts of southern Europe (Portugal, Spain, Italy, Slovenia) to the eastern parts, where it includes three geographically separated subgroups: the northern subgroup (Poland, Ukraine, Romania), the southern subgroup (Albania, Bulgaria) and the central subgroup (Bosnia and Herzegovina, Serbia, Hungary). The Mesoamerican common bean landraces probably arrived in Europe through Spain and Portugal in the year 1506, and the Andean in a similar way in 1528, after the exploration of Peru by Pizarro [7, 49]. The common bean is distributed throughout Europe, Asia and Africa, where it presents similarities to the Mesoamerican and Andean gene pools, and hybrids have formed between these two gene pools [7, 23, 50, 51]. Maras et al. [23] reported that for the western Balkan countries, their Macedonia accessions were evenly spread across both of these gene pools, which might indicate that *P. vulgaris* was introduced into the western Balkans mainly from the Mediterranean basin. With no reference to the number of accessions from each geographic origin, higher levels of genetic diversity were detected within the Hungary, Italy, Poland and Slovenia accessions, considering that *uH*_*e*_ > 0.800 and *Ar* > 4.000. These accessions might therefore be identified for conservation purposes. The highest numbers of genetic clusters (*K* ≥ 9) were seen for Portugal, Serbia, Ukraine, Italy and Poland, which revealed the highest levels of genetic diversity among the accessions that originated from these geographic areas.

On the basis of the genetic structure, the most genetically admixture accessions were from Hungary, where only three genetic clusters were seen. For the PCoA for the Romania accessions, the lower proportion of molecular variability seen via the covariance distance matrix (62.8%) was explained by the first three axes, which reflected the minor significance of the distribution of these accessions, compared to other geographic origins.

For the global analysis using the three *Pv*SHP1 markers, five genetic clusters were seen, which revealed one subgroup for each gene pool (i.e., Mesoamerican, Andean) and a common subgroup that comprised accessions from mixed origins (possible putative hybrids). This finding can be briefly confirmed by the genetic relatedness of the accessions with known phaseolin type, where five groups were also defined by the phylogram obtained. The typical Andean phaseolin type T was distinguished from the Mesoamerican type S with few exceptions (i.e., IT4102T, PHA0107prB, PHA418siT). When specifying the population of geographic origin, the *Pv*SHP1 markers distinguished among the whole collection between the Mesoamerican and Andean gene pools. Moreover, this showed that the Andean accessions are more prevalent than the Mesoamerican ones within some specific geographic origins. The global distributions of the accessions using no prior information revealed three main groups that again represented the Mesoamerican, Andean and mixed groups for the whole western-to-eastern European *P. vulgaris* germplasm. The combination of the *Pv*SHP1 markers was therefore the only efficient analysis to show the detailed gene-pool affiliations of these European accessions (with *Pv*SHP-1B as the most distinguishing locus), especially for the central-eastern part of Europe. In contrast, the *Pv*SHP1-C locus was not informative for the Bulgaria, Albania and Portugal accessions, and similarly, the *Pv*SHP1-A locus was not informative for the Spain accessions. As seen from these data, which *Pv*SHP1 is applied for which geographic origin within the European *P. vulgaris* germplasm is important. Hence, a few well-chosen SSR markers are better for discerning gene-pool affiliations than many SSRs. The geographic origin and informativity/ usefulness of the *Pv*SHP1 markers applied in the present study reflect the data shown in Fig. 4a and in the pie charts in Fig. 4b. Combining all three markers of the European common bean germplasm was useful to distinguish their origins according to the Mesoamerican, Andean and mixed groups. The present study showed that this European common bean germplasm was mostly Andean, with an exception for two of the border countries here: Spain (western Europe) and Ukraine (southern Europe). As suggested by Nanni et al. [9], these three *Pv*SHP1 indel-spanning markers are useful for germplasm identification, and particularly to trace the distributions of the domesticated Mesoamerican and Andean gene pools. These three markers were the molecular markers that highlighted the polymorphism among the different common bean genotypes due to the presence/ absence of indels in the *Pv*SHP1 gene sequence. The data from the three *Pv*SHP1 markers successfully indicated the gene-pool origins of the European accessions (as seen from Fig. 4a, b). As a comparison, when using the whole set of the 33 markers, the global PCoA distribution was extracted in detail considering the geographic origin of each accession from the individual countries (as seen from Fig. 4c). Hence, additional information is presented to show the variability of the accessions among these European countries.

Nowadays, technology allows thousands of SNP markers to be genotyped through many genomes [52–54]. Recently, Blair et al. published a study where describe recombination and linkage disequilibrium in the *P. vulgaris* genome using a 768-marker array of SNPs based on Trans-legume Orthologous Group genes along with an advanced-generation Recombinant Inbred Line reference mapping population (BAT93 x Jalo EEP558) and an internationally available diversity panel [55]. Gene-based (SSR) markers are important for genome-wide association studies [56]. Even though SSRs are still important from a candidate gene approach, they are not in a genome-wide association studies paradigm. In the present study, out of the whole set of 33 SSR markers, six SSR markers were selected as trait/ gene-related markers that are suitable for association mapping studies. These were included in the study of Perseguini et al. [57], who developed a common bean core collection of 500 *P. vulgaris* accessions. For these (with 58 SSRs), the mean *PIC* was only 0.29. In the present study, for the Spain, Slovenia and Italy accessions, the levels of genetic diversity were high due to the highest *uH*_*e*_ and *H*_*e*_ values seen. The highest genetic potential for association mapping studies was calculated for the Hungary, Serbia and Slovenia accessions considering the alleles that were specific for the accessions from each of these geographic origins (i.e., the *Np* values). On the basis of chloroplast markers and two unlinked nuclear loci (*Pv*SHP1), Angioi et al. [58] estimated that a relatively high proportion of the European bean germplasm (about 44%) was derived from hybridisation between the Mesoamerican and Andean gene pools. The data from the present study that was conducted on the basis of nuclear assessments show that the highest proportion of the accessions was of Andean origin. On the other hand, there were indications of subgrouping from the Mesoamerican and Andean gene pools on the basis of the *Pv*SHP1 markers, where five genetic clusters were defined for this western-to-eastern European germplasm. As a comparison, Sinkovič et al. [59] reported that on the basis of only 14 morphological characteristics of the seeds, the *P. vulgaris* and *P. coccineus* germplasm from a gene bank in Slovenia (which also reflects its origin) consisted of three groups.

## Conclusions

The data from the present study show that this collection of 782 accessions that originated along a western-to-eastern line of countries through southern Europe represents a valuable source of genetic variability. These have known backgrounds, which is generally useful for further genetic studies and for formation of a core collection that comprises the most promising accessions from these different geographic origins. Furthermore, we have shown that the 33 genome and gene-specific SSR markers used are highly applicable for diversification studies of European *P. vulgaris* accessions, and have sufficient power to distinguish between and within the Mesoamerican and Andean germplasms. Moreover, three indel-spanning markers of the *Pv*SHP1 gene are shown here to be efficient and informative for determination of the gene-pool affiliations of these European accessions. We have also shown that these European gene banks have successfully maintained, and are sharing, this highly diverse European *P. vulgaris* germplasm for scientific and applicative purposes.

We have shown here that both the Mesoamerican and Andean accessions came to Europe following a western-to-eastern line through southern Europe. During this spread, they adapted to the European agro-climatic environment, which is reflected in the increased diversification levels that include mixed subgroups of Mesoamerican and Andean accessions and alleles that are specific for each geographic origin. However, any conclusions relating to adaptation need to be drawn up with care given the study questions and the analyses that were carried out, whereby the only clues in this regard should come from new analyses. As a perspective of the present study, the use of Approximate Bayesian Computation modelling can be recommended to check the estimates from this study. Another perspective would be the use of newer approaches. Cortes et al. [54] identified 84 gene-based SNP markers, and they detected slightly higher intra-population diversity within the Andean gene pool compared to that within the Mesoamerican gene pool. However, they emphasised that SSR markers are still essential to determine stratification, parental polymorphisms and the evolutionary processes that have occurred within each gene pool [54]. Additionally, Galeano et al. [55] reported on the discovery of SNPs in candidate genes or transcript sequences (i.e., expressed sequence tags), which has been a recurrent strategy in plant genetics mainly because gene-based SNP markers can themselves be causative SNPs for traits that are mainly formed for the Andean diversity panel of *P. vulgaris*. Such gene-based SNP markers from both of these previous studies might be particularly useful for analysis of the European *P. vulgaris* germplasm, where the Andean genotypes are dominant.

## Methods

### Initial screening based on multi-crop passport descriptors

We used the web-based EURISCO for the *Phaseolus* database, with the identification of > 800 accessions that covered diverse environments from different parts of the European continent, following from Spain to Ukraine, along a western-to-eastern line of southern European countries, with Poland included in the sampling. The available data related to basic multi-crop passport descriptors and the seed characteristics were acquired from different national gene-bank curators, including geographic origin, biological status, ancestral data, phenotypic seed characteristics, phaseolin type (corresponding to Mesoamerican/ Andean origins), and seed material.

The plant material was available in limited amounts, with 9 to 32 seeds received for each accession, depending on the gene-bank availability. Out of these > 800 accessions identified, we succeeded in acquiring 782 accessions based on certain gene-bank availability and the viability of the requested accession. Each ‘accession’ represented a population or genotype, which included cultivars, commercial varieties and landraces. The original sources of the plant material were the following gene banks: AIS (Slovenian and Albanian accessions), IBGR-UNI-BL (Bosnia and Herzegovina), CPD (Hungary), CSIC (Spain), DAB (Italy), IHAR (Poland), IPK (Germany), and IRP-NS (Serbia). All of the accessions included in the study are listed in Additional file 4: Table S1. The seed material was transferred via Seed Material Transfer Agreements for research purposes within the L4–7520 Project. These documents are available from the authors.

### Plant material and DNA extraction

Young plants were grown in a greenhouse at the Agricultural Institute of Slovenia (latitude, 46°06′; longitude, 14°51′; altitude, 320 m a.s.l.). Before sowing, six seeds from each accession were chemically disinfected with 5% sodium hypochlorite. At the phase of the first true leaves, 60 mg to 100 mg fresh and healthy plant tissue was sampled. The extraction procedure for DNA was performed as described by Pipan et al. [27, 30] and Maras et al. [23]. The DNA concentrations of each of the isolates were determined using a fluorimeter (Qubit 3.0; ThermoFisher Scientific, MA, USA), with the DNA diluted to the final uniform concentration of 5.6 ng/μL. A *P. coccineus* accession was included as an outgroup accession (i.e., PHA220, from Slovenia). Accessions with available data on phaseolin type were used as internal standards or anchors, to assure the correct gene-pool assignment. These included the Mesoamerican S, M and B phaseolin types, and the Andean T, C and H phaseolin types. The main reason why we used phaseolin-based controls rather than well-known gene pool controls, such as Andean genotypes Calima (G4494) and Chaucha Chuga (G19833), as well as the Mesoamerican genotypes ICA Pijao (G5773) and Dorado (DOR364), is that there was the need to include and analyse accessions that were native to western and eastern European countries (e.g., Slovenia, Italy, Spain, Portugal; see accessions in Fig. 3) from where the other analysed accessions in this study came (i.e., their native geographic environment and origins).

### Genotyping and fragment analysis

To define the diversification and genetic structure of the *P. vulgaris* germplasm, a set of 33 genome-specific markers were used, as developed by nine research groups [9, 37–44]. The criterion of choice was a distribution across all of the linkage groups (i.e., 24 SSR markers). The three indel spanning markers SHP1-A, SHP1-B and SHP1-C were used for identification of the gene pool of origin [9]. Six markers from Benchimol et al., Campos et al. and Hanai et al. were used [40, 41, 43], as these were associated with specific genes (for detailed data, see Additional file 5: Table S2).

The PCR reactions were performed in a final volume of 11.5 μL that contained 8.4 ng genomic DNA and the following reagents, with initial concentrations: 1 μL of 10× PCR buffer (Biotools, Spain), 0.2 μL of mix from each 10 mM dNTP (Sigma-Aldrich, ZDA), 0.5 μL 50 mM MgCl_2_ (Biotools, Spain), 0.1 μL 10 μM forward primer (Sigma-Aldrich, ZDA), 0.25 μL 10 μM reverse primer (Sigma-Aldrich, ZDA), 0.183 μL 10 μM 5′-fluorescently labelled universal primer (with 6-FAM, NED or HEX; Omega, Slovenia), and 0.5 μL 5 U Taq DNA polymerase (Biotools, Spain). The forward primer of each SSR had an added 18-bp tail sequence of 5′-TGTAAAACGACGGCCAGT-3′ (M13(− 21)), as described by Schuelke [60].

The PCR analyses were performed using a thermal cycler (Veriti; ThermoFisher Scientific) under the following ‘touch-down’ conditions, which was dependent on each primer pair: 94 °C for 4 min; 15 cycles at 94 °C for 1 min; decreased temperature from 60 (62) °C to 49.5 (51.5) °C at 0.7 °C per cycle for 30 s; 72 °C for 1 min; followed by 23 cycles at 94 °C for 30 s; 53 °C for 30 s; 72 °C for 1 min; and final extension for 5 min at 72 °C. Fragment analysis was performed on a genetic analyser (3130XL; Applied Biosystems), and allele lengths were determined by comparison with an internal size standard (GeneScan-350 ROX; Applied Biosystems) using the GeneMapper 4.0 software (Applied Biosystems).

### Data analysis

The following parameters were calculated using the Identity 1.0 [61] and Microsatellite-Toolkit [62] software: variability, including observed number of alleles (*No*); expected heterozygosity (*H*_*e*_); and polymorphic information content (*PIC*). The following further parameters were calculated for each of the 12 geographic groups of accessions using the GenAlEx 6.1 software [63]: number of effective (*Ne*) and private (*Np*) alleles; Shannon’s information index (*I*); fixation index (*F*); alleles with frequency > 5%; number of common alleles with proportions of ≤25% and ≤ 50%; unbiased expected heterozygosity (*uH*_*e*_); and locus-specific deviations from the Hardy–Weinberg equilibrium (*HWE*). The same software was used for principal coordinate analysis (PCoA). Analysis of molecular variance (AMOVA) was performed to determine the genetic variation within and among the groups of accessions (geographic origin, gene-bank origin) using the Arlequin software [64]. Due to the application of well-established markers, the frequency of null alleles is not presented, while the amplification was true and unmasked. Preliminary calculations showed that there was no need to present the results about null alleles among the loci. Moreover, other summary statistics for the markers and the population diversity parameters (e.g., Fstatistics, genetic distances, *HWE*, and others) did not show any disturbances that might be influenced by significant levels of null alleles. The estimation of gene flow for each geographic origin was carried out by calculation of the effective numbers of migrants using the private allele method of Slatkin [46] and the Genepop 4.1.0 software [65], which reported the corrected estimated value of Barton and Slatkin [47]. The Populations 1.2.28 software [66] was used for computation of Nei’s standard genetic distances [45] from the allele frequencies, and for construction of the unweighted pair group method with arithmetic mean (UPGMA) dendrogram in the cluster analysis under bootstrapping (100 times). The UPGMA dendrogram was visualised using the TreeView software [67]. The dendrogram was rooted with *P. coccineus*, which was not originally defined as an outgroup as it was treated the same as the *P. vulgaris* accessions, while outgroups give polarity to unrooted trees and allow reconstruction of the ancestral character states and areas [68]. Statgraphics Centurion XVI (2009) was used to define the dendrogram of genetic relatedness between the geographic origins, using squared Euclidian methods and Ward’s algorithm under bootstrapping (100 times). The Structure 2.3.3 software [69] was used to infer the population structure using a Bayesian approach, explained by the posterior probability that each accession belonged to each genetic cluster (*Q* value). Twenty independent runs for each *K* (from 1 to 15) were performed for the admixture model, with a burning period of 10,000 followed by 100,000 Markov chain Monte Carlo repeats. The real *K* value was selected based on the increase in the likelihood ratios between runs, using the Evanno delta *K* statistic [70], and implemented in the Structure Harvester software [71]. An accession was assigned to a specific cluster when its percentage of membership was between 80 and 100%. When using the Structure software, different information for the specifying of the numbers of populations (origins) was used in the input matrices (i.e., without specifying the population or geographic origin [only one/ European origin]; using the information for the gene-bank origins [eight gene-bank origins]; and specifying the geographic origin [12 geographic origins]) under the same criteria for choosing the optimum number of clusters for the different kinds of analysis performed. Global factorial correspondence analysis (FCA) and linkage disequilibrium between the accessions from the different geographic origins were carried out using the Genetix 4.05 software [72], and the allelic richness (*Ar*) for accessions within each geographic origin were obtained using the FSTAT 2.9.3.2 software [73]. Specific datasets were standardised before the calculations where needed, to avoid any impact of set size.

## Supplementary information

**Additional file 1: Figure S1.** Structure plot of the 782 accessions.

**Additional file 2: Figure S2.** Phylogenetic relationships for the complete set of accessions, based on Nei’s standard genetic distances and the UPGMA clustering method.

**Additional file 3: Figure S3.** Genetic structure of the accessions, specifying their population of origin.

**Additional file 4: Table S1.** Accessions used in the present study.

**Additional file 5: Table S2.** SSR markers applied in the present study.

**Additional file 6: Table S3.** Analysis of molecular variance considering the gene-bank origins of the accessions.

**Additional file 7: Table S4.** Loci without statistically significant deviations from *HWE* (*p* > 0.05).

## Data Availability

The datasets supporting the conclusions of this study are included here and the Supplementary Materials.

## Abbreviations

AMOVA: Analysis of molecular variance
*Ar*: Allelic richness
*F*: Fixation index
FCA: Factorial correspondence analysis
*H*_*e*_: Expected heterozygosity
*HWE*: Hardy–Weinberg equilibrium
*I*: Shannon’s information index
*Ne*: Number of effective alleles
*Np*: Number of private alleles
*Pc*: *Phaseolus coccineus*
PCoA: Principal coordinate analysis
*PIC*: Polymorphic information content
*Pv*: *Phaseolus vulgaris*
SSRs: Simple sequence repeats
*uH*_*e*_: Unbiased expected heterozygosity
UPGMA: Unweighted pair group method with arithmetic mean
