# Supplementary figures and images for: Diversification and genetic structure of the western-to-eastern progression of European Phaseolus vulgaris L. germplasm
Source: BMC Plant Biol. 2019 Oct 23;19:442. doi: 10.1186/s12870-019-2051-0 (PMC6813049; doi:10.1186/s12870-019-2051-0)

Figure S1. Structure plot of the 782 accessions.


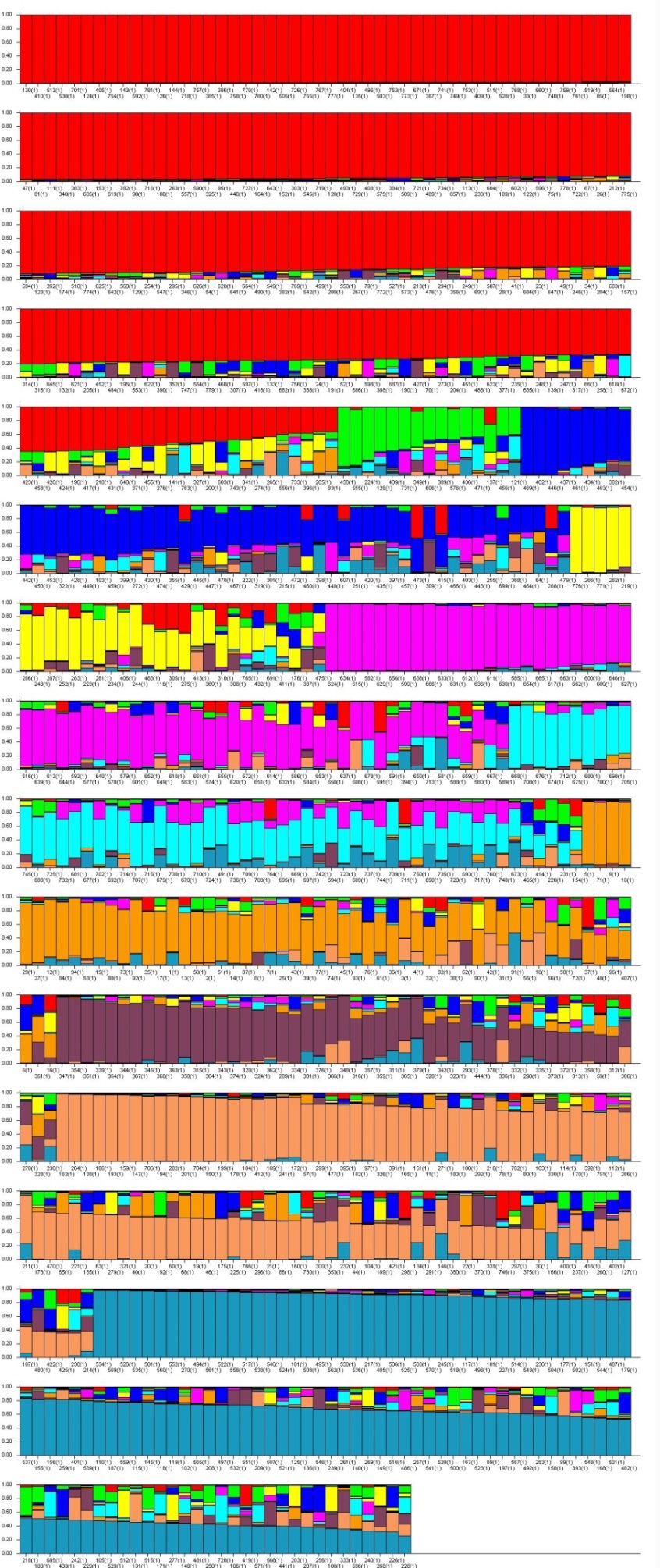

Supplement: Supplementary file 1 — Additional file 1: Figure S1. Structure plot of the 782 accessions. [file 12870_2019_2051_MOESM1_ESM.docx]

Figure S3. Genetic structure of the accessions, specifying the population of origin.
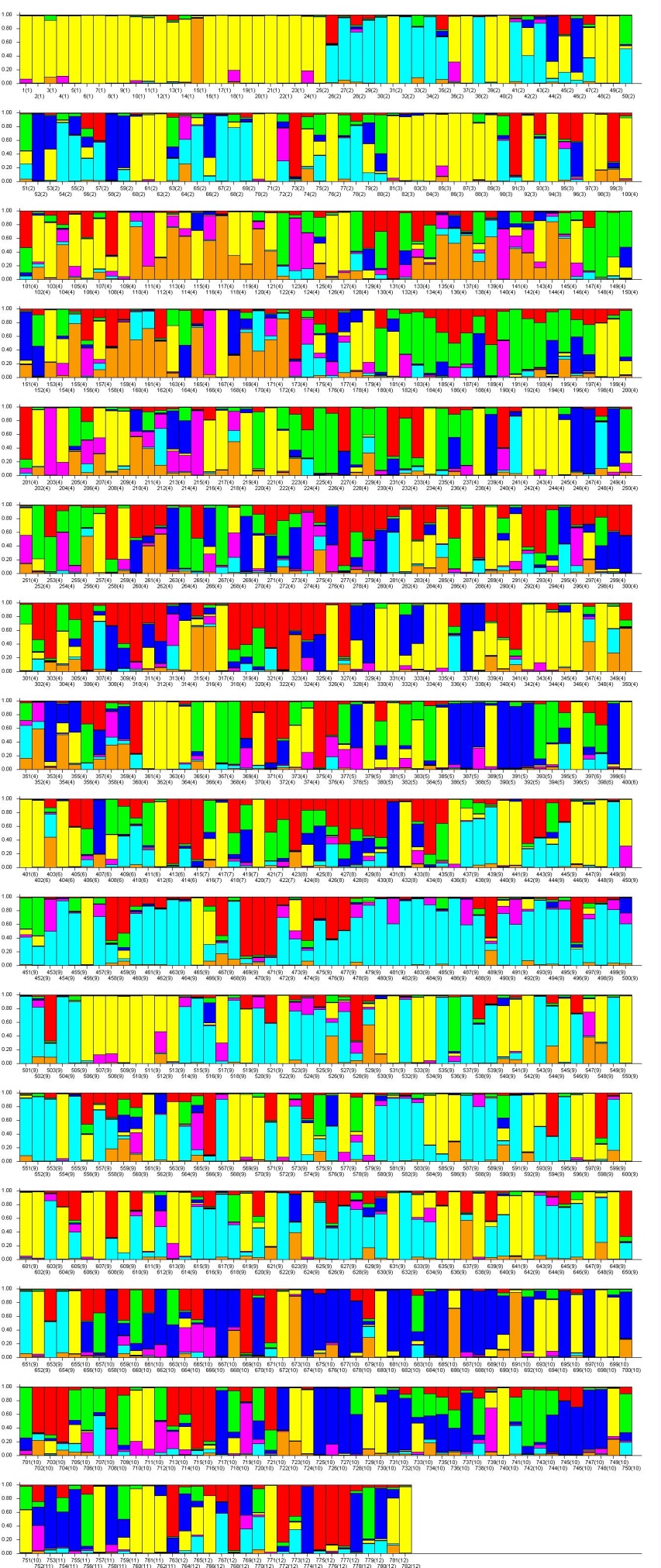

Supplement: Supplementary file 3 — Additional file 3: Figure S3. Genetic structure of the accessions, specifying their population of origin. [file 12870_2019_2051_MOESM3_ESM.docx]
