# Supplementary material for: Diversification and genetic structure of the western-to-eastern progression of European Phaseolus vulgaris L. germplasm
Source: BMC Plant Biol. 2019 Oct 23;19:442. doi: 10.1186/s12870-019-2051-0 (PMC6813049; doi:10.1186/s12870-019-2051-0)
Supplement: Supplementary file 2 — Additional file 2: Figure S2. Phylogenetic relationships for the complete set of accessions, based on Nei’s standard genetic distances and the UPGMA clustering method. [file 12870_2019_2051_MOESM2_ESM.docx]

Figure S2. Phylogenetic relationships for complete set of accessions, based on Nei's standard genetic distances and the UPGMA clustering method.
