# Supplementary material for: Diversification and genetic structure of the western-to-eastern progression of European Phaseolus vulgaris L. germplasm
Source: BMC Plant Biol. 2019 Oct 23;19:442. doi: 10.1186/s12870-019-2051-0 (PMC6813049; doi:10.1186/s12870-019-2051-0)
Supplement: Supplementary file 6 — Additional file 6: Table S3. Analysis of molecular variance considering the gene-bank origins of the accessions. [file 12870_2019_2051_MOESM6_ESM.docx]

**Table S3.** Analysis of molecular variance considering the gene bank origins of the accessions.

| **Source** | **Degrees of freedom (df)** | **Sum of the squared differences** | **Mean square** | **Estimated variance** | **Percentage of molecular variance** |
| --- | --- | --- | --- | --- | --- |
| Among gene bank origins | 8 | 645.187 | 80.648 | 0.449 | 3***** |
| Within gene banks | 1555 | 20557.829 | 13.220 | 13.220 | 97 |
| **Total** | **1563** | **21203.015** |  | **13.670** | **100** |

*p <0.01 (F-statistics)
