# Supplementary material for: Diversification and genetic structure of the western-to-eastern progression of European Phaseolus vulgaris L. germplasm
Source: BMC Plant Biol. 2019 Oct 23;19:442. doi: 10.1186/s12870-019-2051-0 (PMC6813049; doi:10.1186/s12870-019-2051-0)
Supplement: Supplementary file 7 — Additional file 7: Table S4. Loci without statistically significant deviations from HWE (p > 0.05). [file 12870_2019_2051_MOESM7_ESM.docx]

**Table S4.** Loci without statistically significant deviations from HWE (*p >0.05*).

| **Country** | **Loci** |
| --- | --- |
| Albania | ATA004, ATA002, ATA020, *Pv*-ag004 |
| Bosnia and Herzegovina | ATA004, BMd042 |
| Bulgaria | *Pv*SHP1-C |
| Italy | ATA004, ATA002, BMd001, *Pv*-ag004, SSR-IAC16, ATA009, BMd042, *Pv*SHP1-C, *Pv*M21, *Pv*M95 |
| Poland | ATA004, ATA002, *Pv*-ag004, ATA009, BM157, BMd042, ATA289, *Pv*SHP1-C, *Pv*M95, SSR-IAC62 |
| Portugal | ATA004, ATA005, GATS91, ATA002, BM172, BMd001, *Pv*-ag004, BM183, ATA009, ATA006, BMd042, *Pv*SHP1-C, *Pv*M04, *Pv*M21, *Pv*M95, SSR-IAC66 |
| Romania | ATA003, ATA005, ATA002, ATA020, *Pv*-ag004, ATA009, ATA289, *Pv*SHP1-C |
| Spain | ATA005, ATA007, ATA016, ATA002, BMd001, ATA020, *Pv*-ag004, ATA010, BM170, ATA006, BMd042, *Pv*SHP1-C, *Pv*M21, *Pv*M95 |
| Ukraine | ATA004, ATA005, ATA002, BM172, ATA289 |
